# Tobacco Control Progress in Low and Middle Income Countries in Comparison to High Income Countries

**DOI:** 10.3390/ijerph13101039

**Published:** 2016-10-24

**Authors:** Carrie L. Anderson, Heiko Becher, Volker Winkler

**Affiliations:** 1 Institute of Tropical Medicine and International Health, Charité-Universitätsmedizin Berlin, 13353 Berlin, Germany; carrie.l.anderson@gmail.com; 2 Institute of Medical Biometry and Epidemiology, University Medical Center Hamburg-Eppendorf, 20246 Hamburg, Germany; h.becher@uke.de; 3 Institute of Public Health, University of Heidelberg, 69120 Heidelberg, Germany

**Keywords:** smoking prevalence, policy measures, tobacco control, MPOWER, global tobacco epidemic, low income countries, middle income countries

## Abstract

The study aimed to describe worldwide levels and trends of tobacco control policy by comparing low and middle income countries with other income categories from 2007 to 2014 and to analyze the corresponding relation to recent changes in smoking prevalence. Policy measure data representing years 2007 to 2014 were collected from all available World Health Organization (WHO) reports on the global tobacco epidemic. Corresponding policy percentage scores (PS) were calculated based on MPOWER measures. Age-standardized smoking prevalence data for years 2010 and 2015 were collected from the WHO Global Health Observatory Data Repository. Trends of PS were analysed with respect to WHO region and OECD country income category. Scatter plots and regression analysis were used to depict the relationship between tobacco control policy of 2010 and change in smoking prevalence between 2015 and 2010 by sex and income category. Combined PS for all countries increased significantly from 47% in 2007 to 61% by 2014 (*p* < 0.001). When grouped by income category and region, policies were strengthened in all categories, albeit with varying progression. By 2014, tobacco control policy legislation had reached 45% in the Least Developed Countries (LDCs), 59% in Low Middle Income Countries (LMICs), 66% in Upper Middle Income Countries (UMICs) and 70% in High Income Countries (HICs). Overall, there was a negative relationship between higher policy scores and change in smoking prevalence. Although policy strengthening had been conducted between 2007 and 2014, room for considerable global improvement remains, particularly in LDCs.

## 1. Introduction

Tobacco consumption and smoke exposure can have devastating health, social, economic and environmental consequences at both individual and global levels [1]. Although scientific evidence has routinely shown that exposure to tobacco smoke causes death, disease and disability [2], the tobacco epidemic continues to persist at a global scale with a growing emphasis on low and middle income countries. In 2015, it was estimated there were 1 billion smokers globally, with 80% of those living in low and middle income countries [3]. Tobacco is contributing to approximately 6 million deaths each year, with 5 million of those deaths directly attributable to tobacco use [3]. In 2004, tobacco was responsible for 4%, 11% and 18% of total deaths in low, middle and high income countries, respectively [4]. A higher tobacco-related mortality can been seen in men than women, and the American and European regions claim the highest proportion of deaths attributable to tobacco [2]. Nevertheless, tobacco consumption in low and middle income countries is prevalent and such countries continue to confront growing burdens of such modern health risks. A systematic review of smoking prevalence in African adults revealed estimated male smoking prevalence in rural areas up to 22.8% in Rwanda and 40.4% in Zambia [5]. Without implementation and enforcement of proper tobacco control policies, global smoking prevalence could be as high as 22.0% in 2030, with the African region increasing from 15.8% in 2010 to 21.9% in 2030 [6]. The World Health Organization (WHO) predicts the annual death toll caused by tobacco to rise to 8 million by the year 2030 without policy implementation, with over 80% of those deaths in low and middle income countries [7].

In order to standardize global tobacco control policy, the WHO Framework Convention on Tobacco Control (WHO FCTC) treaty was negotiated and brought into fruition. This FCTC treaty was supported by evidence-based tobacco control research conducted in high income countries and other controlled environments [8]. Consisting of 168 signatories, the treaty was unanimously adopted by the World Health Assembly in May 2003 and took effect in February 2005 [1,9]. Created as a response to the expanding globalization of the tobacco epidemic, the treaty encompasses regulatory strategies with legally binding obligations for its parties, which were designed to aid in the protection of public health against the dangers of tobacco usage and against commercial interests of the tobacco industry [1]. Such protection is important in industrialized countries and low and middle income countries as well where tobacco companies continue to exploit markets, deliberately targeting non-smokers, young people and women [10]. This FCTC treaty includes not only tobacco control measures, but also addresses product supply, and demand and harm reduction provisions, providing context for comprehensive policy interventions at all levels of governance.

To assist country level implementation and management of tobacco control articles recommended in the treaty, the MPOWER package was introduced in 2008 consisting of six policy intervention strategies: monitor tobacco use and prevention policies, protect people from tobacco smoke, offer help to quit tobacco use, warn about the dangers of tobacco, enforce bans on tobacco advertising, promotion and sponsorship, and raise taxes on tobacco [7]. Each intervention reflects one or more provisions described in the treaty. Additionally, the treaty encourages countries to develop surveillance programmes to monitor and collect data regarding magnitude, patterns, determinants and consequences of tobacco exposure and consumption, facilitating data comparison at both regional and international levels [1].

Although there is evidence pointing to the effectiveness of tobacco control policies in reducing smoking prevalence in many high and some middle income countries [11], there is limited data on the effectiveness of such policies in low and middle income countries. Furthermore, FCTC research gaps have identified a need to decipher which policies may be effectively transferred and implemented from High Income Countries (HICs) to Low Middle Income Countries (LMICs), and have also suggested a necessity for the acquisition of both country-specific data and to identify further research needs [8].

For the primary aim of this study, we analysed levels and trends of tobacco control policy indicator variables between 2007 and 2014 by country income category and region, according to MPOWER data and with a focus on low and middle income countries. For the secondary study aim, we examined the association between tobacco control policy and the change in current tobacco smoking by an ecological study.

## 2. Materials and Methods

Policy data was extracted from all available WHO reports on the global tobacco epidemic for years 2007 [7], 2008 [12], 2010 [13], 2012 [14] and 2014 [15]. Indicators were extracted and used to generate policy percentage scores (PS) as percentages of the maximum policy defined as fulfilling criteria included in the respective WHO reports.

- *Tax*: In accordance with WHO recommendations [16], national tax values of ≥70% of retail prices were considered as 100% legislated policy. PS was calculated based on tax values scaled to this maximum value. Tax values are based on excise taxes, import duties, value-added taxes (VAT) and other taxes as applicable for the price of the most sold brand. When two non-equal tax values were presented per country and year (i.e., China and Vietnam), averages of the two values were used.
- *Ban on advertising*: PS were calculated as percentages of applying the following categories of bans on direct advertising: national TV and radio, international TV and radio, local magazines and newspapers, international magazines and newspapers, billboard and outdoor advertising, point of sale and internet. For example, in 2008, Bangladesh had direct advertising bans on national TV/radio, local magazines/newspapers and billboard/outdoor advertising, and was assigned a PS of 43% (3/7). In addition, 2014 data included an additional category: fines for violations of bans on direct advertising.
- *Ban on promotion and sponsorship*: PS were based on the following categories of bans on promotion and sponsorship: free distribution by mail or through other means, promotional discounts, non-tobacco products identified with tobacco brand names, brand name of non-tobacco products used for tobacco product, appearance of tobacco products in TV and/or films, sponsored events and product placement (not included for 2007). Furthermore, 2014 data included an eighth category: fines for violations of bans on promotion and sponsorship.
- *Smoke-free environments*: PS were defined as percentages of the number of the following public places with smoke-free legislation: healthcare facilities, educational facilities (excluding universities), universities, government facilities, indoor offices, restaurants, pubs and bars, public transportation (not included for 2007), and all other indoor public places.
- *Availability of cessation support (Support for treatment of tobacco dependence)*: PS were calculated based upon the availability of tobacco dependence support methods. 2007 policy data included three methods of support: a toll-free quit line, nicotine replacement therapy (NRT), and the pharmaceutical smoking cessation aid Bupropion. In addition, 2008 and 2010 data included the pharmaceutical Varenicline, whereas 2012 and 2014 presented data solely on toll-free quit lines and NRT.
- *Regulation on packaging*: The WHO FCTC recommends tobacco packages to carry health warnings that cover ≥50% of the display area [1]. Packaging covered with ≥50% of health warnings were regarded as having 100% legislated policy and thus were assigned a PS of 100%.
- *Existence of Government Objectives on Tobacco Control*: PS was defined as a binary indicator (0% or 100%), indicating whether or not national governments have objectives on tobacco control in place.

A combined PS was calculated for each year and country as the arithmetic mean of all collected indicators as similarly done by Dubray et al. [17].

Income category was assigned based on the Organization for Economic Co-operation and Development (OECD) [18]. Countries with a 2013 per capita gross national income (GNI) of ≤$1,045 and Least Developed Countries (LDCs) as defined by the United Nations (UN) were combined to be categorized as Least Developed Countries, a GNI of $1,046–$4,125 as Lower Middle Income Countries (LMICs), a GNI of $4,126–$12,745 as Upper Middle Income Countries (UMICs), and a GNI of ≥$12,746 as High Income Countries (HICs).

Countries were grouped into the following six geographical regions according to WHO’s classification system: Africa, Eastern Mediterranean, Europe, South-East Asia, the Americas and Western Pacific [19].

Age-standardized (per WHO standard population) and sex-specific current smoking prevalence estimates per country for adults ≥15 years were collected for years 2010 and 2015 from the WHO Global Health Observatory Data Repository [20]. “Current tobacco smoking” includes daily, non-daily and occasional smoking of cigarettes, cigars, pipes, or any other smoked tobacco product, excluding smokeless tobacco [20]. Estimates used had been derived using data obtained by specific population surveys and surveillance systems and a statistical model based on Bayesian negative binomial meta-regression. Details regarding the methodology for estimating smoking prevalence are described by Bilano et al. [21]. Countries were excluded from the data analysis if measured indicators for a particular year were missing or unavailable (39, 15, 22, 11, and nine countries for years 2007, 2008, 2010, 2012 and 2014), indicating an inability to calculate a combined PS. 

Descriptive analysis of all indicators including the combined PS was conducted over time by (i) year and income category; and (ii) year and region.

Additionally, scatter plots were used to depict the relationship between the combined PS of 2010 and change in current tobacco smoking between 2015 and 2010 (difference prevalence 2015 minus prevalence 2010) in relation to smoking prevalence in 2010 by sex and income category. Data from 2010 was selected as the baseline due to a lesser number of missing values, and the period between 2010–2015 was selected to provide ample time to reflect the influence of such policy changes. For visualization, the combined PS was categorized into four groups according to percent policy legislation: <40%, 40%–59.9%, 60%–79.9% and ≥80%. Linear regression was used to model the difference of smoking prevalence in 2015 and 2010 (dependent variable) as a function of PS of 2010 (continuous) stratified by sex while controlling for smoking prevalence in 2010 and income category (categorical; independent variables). 

Both policy and prevalence scores were coded as XX.X. *p*-values refer to the linear trend for year and are derived from linear regression using policy indicator as the dependent and year as the independent variable. A *p*-value < 0.05 was used for significance.

StataIC version 14.0 (StataCorp, College Station, TX, USA) was used for data processing and analysis.

## 3. Results

### 3.1. Trends of Tobacco Control Policy

Information on study data and progression of policy indicators over time by country income category are presented in Table 1. Information with regard to region is shown in Appendix A (Table A1). It was discovered that 181 to 195 countries were available from the WHO reports. With regard to income category, data pertaining to all measured indicators were complete for 85%, 95%, 90% and 92% of the LDCs, LMICs, UMICs and HICs, respectively. Geographically, most missing values were from the Eastern Mediterranean region. Data pertaining to all measured indicators were complete for 94%, 90%, 88%, 78%, 91% and 89% of countries in the African, Eastern Mediterranean, European, South-East Asian, American and Western Pacific regions, respectively.

**Figure 1:**
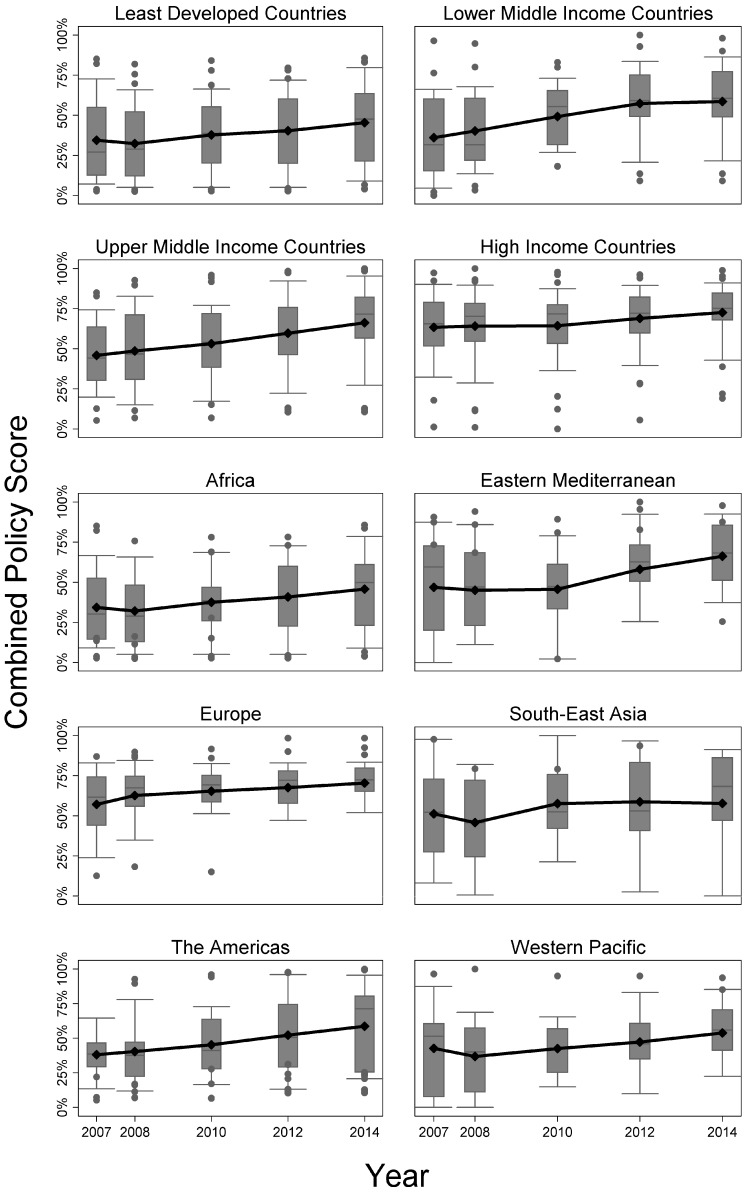
Progression over time of the calculated combined policy score by (i) income category and report year (**top** four panels) and by (ii) World Health Organization (WHO) geographical region and year (**bottom** six panels).

Progression over time of calculated combined policy score by income category and by region is shown in Figure 1. PS, policy percentage scores.

Overall, PS significantly increased from 47% in 2007 to 61% by 2014 (*p* < 0.001). By income category, a similar trend of increasing PS was observed, which was significant for all levels. HICs had the smallest increase of 13% but overall the highest PS of 70% in 2014 (*p* = 0.012). PS of LDCs increased by 32%, reaching 45% in 2014 (*p* = 0.006). UMICs showed the highest increase over time (43%), raised from 46% to 66% (*p* < 0.001).

Furthermore, each region maintained the trend of increasing PS, which was significant overall (*p* ≤ 0.001) and in each region with the exception of South-East Asia (*p* = 0.402). The Americas had the largest increase in mean PS of 55%, whereas South-East Asia had the lowest increase in PS of 7%. Final 2014 PS for Africa resulted in 46%, and the highest score of 71% was observed in Europe.

Overall, most policy indicators strengthened over time, despite differences in income category or region. However, several indicators presented with opposite effects, decreasing between 2007 and 2014. Tax in the Eastern Mediterranean and South-East Asia; promotion in HICs and South-East Asia; smoke-free areas in LDCs, HICs and Africa; cessation support in LMICs, HICs, Africa, Europe and the Western Pacific each dropped a few percentage points over time, hence showing weakening of respective tobacco control policies over time.

### 3.2. Association between Tobacco Control Policy and Change in Smoking Prevalence

**Figure 2:**
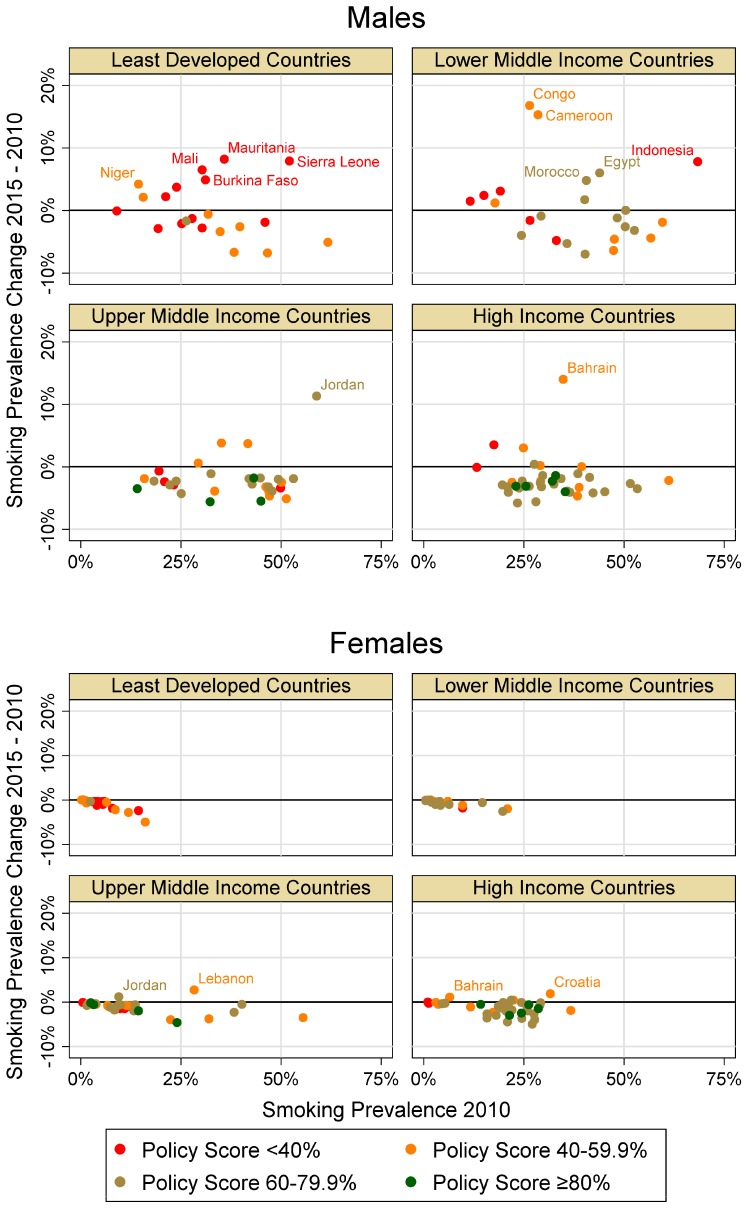
Relationship between combined policy percentage scores (PS) of 2010 and change in current tobacco smoking between 2010 and 2015 in relation to smoking prevalence in 2010 by gender and country income category (labeled data points indicate countries with a smoking prevalence change of >4% for males and >1% for females; Congo = Republic of Congo).

A graphical representation of the relation of combined PS of 2010 and change in current tobacco smoking between 2010 and 2015 in relation to smoking prevalence in 2010 by sex and country income category is depicted in Figure 2.

Of all countries included in years 2010 and 2015, age-adjusted smoking prevalence data was available in 117 countries (61%) for females, and in 115 countries (60%) for males. Estimates were missing for 48%, 25%, 38%, and 15% of LDC, LMIC, UMIC and HIC countries, respectively. Regionally, data for 37% of countries in the African, 57% in the Eastern Mediterranean, 7% in European, 12% in South-East Asian, 47% in the American and 30% in the Western Pacific were missing. For all countries combined, mean prevalence decreased from 2010 to 2015 by 1.1 percentage points for both females and males. Overall mean prevalence was approximately three times higher for males than females.

When separated by income category, female prevalence decreased between 0.6 and 1.5 percentage points. Male prevalence in LDCs and LMICs increased slightly by 0.1 and 0.5 respectively, whereas prevalence in UMICs and HICs each decreased by two percentage points.

For all countries, male smoking prevalence increased more frequently and of a greater magnitude than for females. From 2010 to 2015, male smoking prevalence increased >10% in four countries: Cameroon and Congo in LMICs; Jordan in UMICs; and Bahrain in HICs. Additionally, male smoking prevalence increased from 2010 to 2015 in LDCs predominately in countries with low PS <40%.

Few countries showed an increase in male smoking prevalence despite a combined PS of >60% i.e., Morocco and Egypt in LMICs; and Jordan in UMICs. A combined PS of >45% corresponded with an increase in female smoking prevalence in Jordan and Lebanon in UMICs; and Croatia and Bahrain in HICs.

Overall, there was a negative relationship between the 2010 policy score and the difference of smoking prevalence in 2015 and 2010 for males and females when controlling for the baseline prevalence in 2010 and income category (Table 2). However, the relationship was stronger in males than females and significant only in males. Model 2 additionally includes the interaction term between income status and policy score (*p* = 0.004, significant only in males) showing policy score impacts differently according to income status.

## 4. Discussion

Overall, the number of legislated WHO policy recommendations for tobacco control increased globally from 2007 to 2014 with the largest increases observed in LMICs and UMICs. Although LDCs steadily strengthened policy over time, by 2014, only 45% of the recommended policies were legislated, similar to the levels of LMICs and UMICs in 2007. In 2014, HICs had the highest number of legislated policies of 70%. The African region improved policy, but remains far behind all other regions. Regression modelling showed a negative association between WHO recommended policy legislations and increase in smoking prevalence, which was stronger in males than females and only significant in males.

Although some country specific smoking prevalence reductions have occurred during the past decade, improvements are nonhomogeneous and around 50 million people, mostly males in LDCs and LMICs, have lost their lives due to tobacco usage [22]. This visible discrepancy between treaty introduction and remaining high tobacco associated mortality most likely cannot be explained simply, but rather by a multitude of intertwined factors. However, it is of utmost importance to consider that MPOWER indicator data represent policy legislation and do not necessarily reflect policy implementation. The true degree of implementation and policy enforcement could vary heavily and be dependent upon per capita GNI, as tobacco control policies stagnate at low levels of implementation in most LMICs [23]. Some countries may have a limited capacity for enforcement, also suffering from the tobacco industry’s heavy influence on local government officials, brand-stretching and the presence of cross-border marketing activities [24,25]. A 2013 review pertaining to tobacco control in LMICs discussed the necessity of national level political economy analyses to explore which barriers may be present in tobacco control and the importance of developing a plan against them [23]. While there may be a strong relation between the number of legislated policies and enforced policies, it remains clear that, in practice, enforcement is never perfect. In other words, our study overestimates the strength of tobacco control policy by exploring national lawmaking only.

Our study supports results reported by other research groups in the area of MPOWER policy relating to smoking prevalence. Recent research by Dubray et al. analysed the relationship between the change in current smoking between 2006 and 2009 with a 2008 MPOWER score [17]. Although most estimates were from HIC and UMIC, it was reported that overall countries with higher policy scores tended to have greater decreases in current tobacco smoking between 2006 and 2009.

Another study by Heydari et al. looked at 2008 MPOWER data to provide an overview of policy in the Eastern Mediterranean region, concluding that three of 21 countries scored higher than 50%, while over half of the countries scored <26% [26]. Our data and other evidence by Heydari et al. suggest that although some progressive policy strengthening has happened in the Eastern Mediterranean, country improvements are not homogeneous and even some policy waning has occurred [27]. Recent estimations by Bilano et al. predicted rapid increases in smoking prevalence by 2025 for males in Africa and both genders in the Eastern Mediterranean [21]. Corresponding strengthening of policies could help prevent smoking, hence mitigating the burden of smoking related diseases in such LMICs in early stages of the tobacco epidemic. Bans on tobacco advertising, promotion and sponsorship (TAPS) have been realized to be important in developed countries, but evidence has been accumulating to show the importance of comprehensive TAPS policies in developing countries [28]. Blecher et al. concluded that comprehensive advertising bans in developing countries are more effective in reducing tobacco consumption than partial bans. Such bans may have a larger impact on tobacco consumption in developing countries relative to the developed world as the number of tobacco advertisements was determined to be 81 times higher in selected low income countries compared relative to HIC [29]. A previous study relating cigarette affordability and consumption showed decreased cigarette consumption in HIC relative to lower income countries that could not be fully explained by changes in affordability [30]. Strong FCTC policy interventions in HICs were postulated to be responsible for decreases in consumption, whereas the social acceptability of smoking and weaker policy interventions could explain the higher tobacco consumption in LMICs. Tobacco price does not equate to affordability. Although tobacco price is typically higher in HICs than in UMICs, LMICs and LDCs, cigarettes are more affordable in HICs [30]. However, evidence is inconclusive regarding whether demand for tobacco products in LDCs is more responsive to price than in HICs [31]. Nevertheless, it has been recommended that governments should consider increasing excise taxes on tobacco to limit consumption and decrease prevalence of current smoking [31]. As tax and price policies constitute highly effective means in influencing tobacco demand and consumption, implementing such strategies as outlined in Article 6 of the FCTC remains of imperative importance in battling the tobacco epidemic [1].

Despite FCTC legislation, global presence of tobacco industries undermines the effectiveness of tobacco control policy. Tobacco companies have been known to employ strategies to evade marketing restrictions by influencing government officials to deter policy making, leading to the maximization of marketing opportunities [24]. Partial advertising bans can be effectively circumvented by the industry targeting non-banned and non-implemented advertising avenues; whereas cross-border advertising can be targeted in countries where full bans have been implemented [24]. Due to decreasing tobacco consumption in HICs, industries may target markets in LDCs and LMICs in the Asian-Pacific, African and Middle Eastern regions where consumption is currently on the rise [10], with Africa as the continent predicted to have the highest increases in smoking prevalence [6,21]. Although gender gaps have narrowed regarding smoking prevalence in most HICs and parts of Latin America and Eastern Europe, female smoking prevalence remains lower than male smoking prevalence in many LDCs and LMICs, encouraging industrial expansion into formerly untapped markets—recruiting non-smokers, women and children in LDCs and LMICs [5,10,25]. A study investigating gender empowerment and female-to-male smoking prevalence ratios (female empowerment as measured by economic participation and decision-making, political participation and decision-making, and power over economic resources), determined that rates of female smoking were higher than males in countries with higher female empowerment [32]. Estimations have suggested higher levels of male smoking (in some LDCs and LMICs) can help mitigate restrictions on female smoking, hence encouraging the habit in females [33]. The marketing of tobacco company products in LMICs combined with bolder industrial attempts to influence political activity and misrepresent the economic importance of tobacco [10], may increase overall smoking prevalence. Together, these research findings indicate an implicit need for heightened tobacco control strategies focussed not only on male, but also on female prevention.

As an ecologic study this analysis has several limitations. Policy data from WHO reports and estimates on smoking prevalence were unavailable for some countries. Additionally, MPOWER policy data reflect the presence of policy legislation and not implementation. Furthermore, the indicator “Existence of Government Objectives on Tobacco Control” was exclusively available as a “yes/no” indicator and therefore lacks sensitivity. The definition of some policy indicators e.g., “Availability of Cessation support” slightly changed by report year which may have led to some bias within policy estimates over time. Nevertheless, we are aware of those limitations and addressed them if possible. Our main intention was to incorporate as many of the globally available data as possible. Finally, income category and region are highly correlated, i.e., many African countries were contemporaneously categorized as LDCs, many European countries were HICs and vice versa. Therefore, it is not possible to estimate independent effects of income category and region.

## 5. Conclusions

Although noticeable progress in strengthening tobacco control policy has been made, room for considerable improvement remains for all categories, particularly in the LDCs. General trends of decreasing smoking prevalence were observed with increasing MPOWER policy scores for both genders, which was stronger in males than in females, and only significant in males. Further research pertaining to which non-MPOWER measures are associated with both increases and decreases in smoking prevalence are recommended, as well as investigation regarding trends influencing tobacco policy adoption, implementation and enforcement within the different global income categories and regions.

## Figures and Tables

**Table 1:** Trends of tobacco control policy by country income category and year. Measurements as ‘*n* or mean% (range)’ as indicated. Per income category, full range (0%–100%) assumed unless otherwise indicated.

	2007	2008	2010	2012	2014	*p*-Value for Temporal Trend
**Income Category**, *n* (# with combined PS)	**181 (142)**	**194 (177)**	**194 (170)**	**194 (181)**	**195 (185)**	
-Least Developed Countries	47 (36)	51 (42)	51 (41)	51 (47)	52 (47)	
-Lower Middle Income Countries	32 (28)	34 (33)	34 (32)	34 (33)	34 (33)	
-Upper Middle Income Countries	53 (38)	57 (56)	57 (51)	57 (54)	57 (55)	
-High Income Countries	49 (40)	52 (46)	52 (46)	52 (47)	52 (50)	
**Tax**, mean% (range)	**59 (3–100)**	**66 (3–100)**	**68 (3–100)**	**68 (4–100)**	**70 (0–100)**	**0.002**
*By Income Category*						
-Least Developed Countries	47 (3–100)	49 (10–100)	49 (5–100)	48 (4–100)	49 (0–100)	0.875
-Lower Middle Income Countries	49 (11–90)	57 (22–100)	60 (22–100)	61 (23–100)	65 (23–100)	**0.011**
-Upper Middle Income Countries	59 (3–100)	69 (3–100)	72 (3–100)	74 (4–100)	76 (7–100)	**0.003**
-High Income Countries	76 (14–99)	85 (26–100)	85 (20–100)	84 (29–100)	85 (28–100)	0.164
**Advertising**, mean% (range)	**36 (0–100)**	**39 (0–100)**	**41 (0–100)**	**57 (0–100)**	**62 (0–100)**	**0.000**
*By Income Category*						
-Least Developed Countries	33	34	36	48	52	**0.002**
-Lower Middle Income Countries	32	35	41	55	60	**<0.001**
-Upper Middle Income Countries	37	40	41	61	69	**<0.001**
-High Income Countries	41 (0–71)	45 (0–86)	47	62	66	**<0.001**
**Promotion**, mean% (range)	**35 (0–100)**	**41 (0–100)**	**39 (0–100)**	**41 (0–100)**	**45 (0–100)**	**0.021**
*By Income Category*						
-Least Developed Countries	32	35	33	33	38	0.632
-Lower Middle Income Countries	27	31	38	40	44	**0.028**
-Upper Middle Income Countries	31	43	38	48	52	**0.006**
-High Income Countries	48	50	45	43	45	0.400
**Smoke free area**, mean% (range )	**36 (0–100)**	**28 (0–100)**	**35 (0–100)**	**40 (0–100)**	**43 (0–100)**	**<0.001**
*By Income Category*						
-Least Developed Countries	36	23	25	25	31	0.830
-Lower Middle Income Countries	30 (0–88)	30	41	50	50	**<0.001**
-Upper Middle Income Countries	30	30	40	49	55	**<0.001**
-High Income Countries	45	29	34	37	39	0.986
**Packaging**, mean% (range)	**34 (0–100)**	**38 (0–100)**	**45 (0–100)**	**53 (0–100)**	**59 (0–100)**	**<0.001**
*By Income Category*						
-Least Developed Countries	21	19	27	33	42	**<0.001**
-Lower Middle Income Countries	33	37	42	54	60	**0.001**
-Upper Middle Income Countries	32	42	49	56	64	**<0.001**
-High Income Countries	48	54	59	69	70	**<0.001**
**Cessation**, mean% (range)	**51 (0–100)**	**48 (0–100)**	**54 (0–100)**	**53 (0–100)**	**52 (0–100)**	0.445
*By Income Category*						
-Least Developed Countries	20	16	23	23	23	0.291
-Lower Middle Income Countries	48	46	54	51	46	0.944
-Upper Middle Income Countries	53	49	55	53	57	0.332
-High Income Countries	81	82	82	83	78	0.585
**Governmental Objectives on Tobacco Control**, mean% (range)	**57 (0–100)**	**69 (0–100)**	**73 (0–100)**	**77 (0–100)**	**89 (0–100)**	**<0.001**
*By Income Category*						
-Least Developed Countries	38	53	59	67	78	**<0.001**
-Lower Middle Income Countries	68	76	85	88	91	**0.007**
-Upper Middle Income Countries	53	70	75	73	88	**<0.001**
-High Income Countries	72	79	79	83	98	**0.001**
**Combined policy score**, mean% (range)	**47 (3–91)**	**48 (2–94)**	**52 (3–96)**	**56 (3–98)**	**61 (4–100)**	**<0.001**
*By Income Category*						
-Least Developed Countries	34 (3–85)	32 (2–82)	38 (3–84)	40 (3–80)	45 (4–86)	**0.006**
-Lower Middle Income Countries	42 (14–89)	45 (16–87)	52 (28–78)	58 (21–92)	59 (21–90)	**<0.001**
-Upper Middle Income Countries	46 (5–85)	49 (7–93)	53 (7–96)	60 (10–98)	66 (11–100)	**<0.001**
-High Income Countries	62 (7–91)	62 (7–94)	63 (6–92)	67 (11–90)	70 (23–93)	**0.012**

Bold values is the significant *p*-values.

**Table 2:** Linear regression models: difference of smoking prevalence in 2015 and 2010 (dependent variable); Policy score of 2010 (continuous), smoking prevalence in 2010 and income category (categorical; independent variables) by sex.

	Model 1				Model 2 (with Interaction Term)			
	Males		Females		Males		Females	
	β	*p*-Value	β	*p*-Value	β	*p*-Value	β	*p*-Value
PS 2010	−0.037	0.013	−0.007	0.077	−0.027	0.111	−0.006	0.181
Income category		0.072		0.424		0.027		0.970
LDCs	1.468		−0.441		6.136		−0.208	
LMICs	2.561		−0.002		4.443		−0.254	
UMICs	0.295		−0.011		−0.470		0.114	
HICs	ref		ref		ref		ref	
Income category × PS 2010	-		-			0.004		0.132
LDCs					−0.123		−0.007	
LMICs					−0.035		0.004	
UMICs					0.010		−0.002	
HICs					ref		ref	
Smoking Prev 2010	0.001	0.013	−0.053	<0.001	−0.130	0.663	−0.055	<0.001

PS, policy percentage scores; LDCs, Least Developed Countries; LMICs, Low Middle Income Countries; UMICs, Upper Middle Income Countries; HICs, High Income Countries.

## Appendix A

**Table A1:** Trends of tobacco control policy by geographical region and year. Measurements as ‘*n* or mean% (range)’ as indicated. Per region, full range (0%–100%) assumed unless otherwise indicated.

	2007	2008	2010	2012	2014	*p*-Value for Temporal Trend
**Region**, *n* (# with combined PS)	**181 (142)**	**194 (177)**	**194 (170)**	**194 (181)**	**195 (185)**	
-Africa	46 (41)	46 (46)	46 (42)	46 (44)	47 (45)	
-Eastern Mediterranean	22 (15)	22 (21)	22 (21)	22 (22)	22 (20)	
-Europe	51 (39)	53 (45)	53 (43)	53 (47)	53 (51)	
-South-East Asia	11 (8)	11 (8)	11 (8)	11 (9)	11 (10)	
-The Americas	35 (27)	35 (34)	35 (33)	35 (33)	35 (33)	
-Western Pacific	16 (12)	27 (23)	27 (23)	27 (26)	27 (26)	
**Tax**, mean% (range)	**59 (3–100)**	**66 (3–100)**	**68 (3–100)**	**68 (4–100)**	**70 (0–100)**	**0.002**
*By Region*						
-Africa	46 (3–100)	50 (10–100)	51 (16–100)	50 (12–100)	52 (12–100)	0.407
-Eastern Mediterranean	59 (3–97)	54 (3–100)	56 (3–100)	54 (4–100)	57 (4–100)	0.988
-Europe	72 (11–99)	85 (28–100)	87 (29–100)	88 (27–100)	89 (25–100)	**0.001**
-South-East Asia	76 (31–100)	68 (35–100)	70 (36–100)	74 (43–100)	72 (0–100)	0.977
-The Americas	51 (3–100)	64 (22–100)	66 (20–100)	67 (23–100)	67 (23–100)	**0.036**
-Western Pacific	62 (13–100)	72 (17–100)	72 (20–100)	71 (21–100)	74 (4–100)	0.329
**Advertising**, mean% (range)	**36 (0–100)**	**39 (0–100)**	**41 (0–100)**	**57 (0–100)**	**62 (0–100)**	**<0.001**
*By Region*						
-Africa	37	33	34	45	52	**0.011**
-Eastern Mediterranean	55	56	56	75	78	**0.004**
-Europe	41 (0–71)	49	50	69	74	**<0.001**
-South-East Asia	49	49	53	62	66	0.193
-The Americas	9 (0–86)	16	22	37	43	**<0.001**
-Western Pacific	45	41	44	60	66	**0.001**
**Promotion**, mean% (range)	**35 (0–100)**	**41 (0–100)**	**39 (0–100)**	**41 (0–100)**	**45 (0–100)**	**0.021**
*By Region*						
-Africa	29	30	29	30	36	0.454
-Eastern Mediterranean	58	61	66	66	66	0.398
-Europe	42	51	45	47	50	0.500
-South-East Asia	55	56	64	53	53	0.862
-The Americas	10	20	19	27	35	**0.002**
-Western Pacific	42 (0–83)	43	36 (0–86)	43	45	0.650
**Smoke free area**, mean% (range )	**36 (0–100)**	**28 (0–100)**	**35 (0–100)**	**40 (0–100)**	**43 (0–100)**	**<0.001**
*By Region*						
-Africa	36	21	25	27	30	0.998
-Eastern Mediterranean	47	44	38 (0–89)	48	50	0.619
-Europe	41	31	39	40	43	0.276
-South-East Asia	45	36	54 (11–89)	56 (11–89)	58 (11–89)	0.109
-The Americas	19	25	36	45	52	**<0.001**
-Western Pacific	34	22	30	41	45	**0.020**
**Packaging**, mean% (range)	**34 (0–100)**	**38 (0–100)**	**45 (0–100)**	**53 (0–100)**	**60 (0–100)**	**<0.001**
*By Region*						
-Africa	18	19	25	34	46	**<0.001**
-Eastern Mediterranean	36	31	32	59	64	**0.002**
-Europe	44 (0–80)	57	67	65	67	**0.000**
-South-East Asia	34	24	26	49	53	0.084
-The Americas	32	37	42	52	58	**0.006**
-Western Pacific	46	47	57	59	68	**0.023**
**Cessation**, mean% (range)	**51 (0–100)**	**48 (0–100)**	**54 (0–100)**	**53 (0–100)**	**52 (0–100)**	0.445
*By Region*						
-Africa	30	24	33	28	29	0.785
-Eastern Mediterranean	33 (0–67)	47 (0–75)	43	55	50	**0.045**
-Europe	76	73	75	76	71	0.641
-South-East Asia	27 (0–67)	23 (0–75)	30 (0–75)	27	32	0.636
-The Americas	62	59	66	59	63	0.928
-Western Pacific	53	40	50	48	46	0.921
**Governmental Objectives on Tobacco Control**, mean% (range)	**57 (0–100)**	**69 (0–100)**	**73 (0–100)**	**77 (0–100)**	**89 (0–100)**	**<0.001**
*By Region*						
-Africa	37	48	57	63	76	**<0.001**
-Eastern Mediterranean	73	77	82	82	91	0.127
-Europe	59	77	82	83	94	**<0.001**
-South-East Asia	73	82	82	91	100	0.062
-The Americas	54	57	60	66	83	**0.009**
-Western Pacific	80	93	93	93	100	0.057
**Policy score**, mean% (range)	**47 (3–91)**	**48 (2–94)**	**52 (3–96)**	**56 (3–98)**	**61 (4–100)**	**<0.001**
*By Region*						
-Africa	34 (3–85)	32 (2–84)	38 (3–78)	41 (3–88)	46 (4–88)	**0.003**
-Eastern Mediterranean	55 (15–85)	54 (5–87)	54 (5–84)	63 (12–92)	68 (32–90)	**0.009**
-Europe	57 (13–87)	63 (18–90)	65 (15–92)	68 (19–98)	71 (21–98)	**<0.001**
-South-East Asia	57 (27–89)	53 (21–78)	61 (36–91)	62 (23–88)	61 (19–88)	0.402
-The Americas	38 (5–71)	40 (7–93)	45 (6–96)	52 (10–98)	59 (11–100)	**<0.001**
-Western Pacific	56 (21–91)	53 (18–94)	56 (31–90)	59 (25–90)	64 (36–89)	**0.026**

PS, policy percentage scores, bold values is the significant *p*-values.
